# NEOADJUVANT TREATMENT OF LIVER METASTASES OF COLORECTAL CANCER: PREDICTIVE FACTORS OF PATHOLOGICAL RESPONSE

**DOI:** 10.1590/0102-6720202400036e1829

**Published:** 2024-10-28

**Authors:** Nayssem KHESSAIRI, Ines MALLEK, Mehdi MBAREK, Elmontassar Belleh ZAAFOURI, Lassaad GHARBI, Ahlem Lahmar BOUFAROUA, Dhouha BACHA, Sana BEN-SLAMA

**Affiliations:** 1 Salah Azaiz Institute, Surgical Oncology Department – Tunis, Tunísia;; 2 University of Tunis El-Manar, Faculty of Medicine – Tunis, Tunísia;; 3 Mongi Slim University Hospital, Pathology Department – La Marsa, Tunis, Tunísia;; 4 Mongi Slim University Hospital, Digestive Surgery Department – La Marsa, Tunis, Tunísia.

**Keywords:** Colorectal Neoplasms, Neoplasm Metastasis, Liver, Prognosis, Neoadjuvant Therapy, Neoplasias Colorretais, Metástase Neoplásica, Fígado, Prognóstico, Terapia Neoadjuvante

## Abstract

**BACKGROUND:**

Surgery after neoadjuvant chemotherapy (CT) improves the prognosis of colorectal liver metastases (CRLM).

**AIMS:**

The aim of this study was to evaluate the predictive factors of the histological response of CRLM after neoadjuvant treatment.

**METHODS:**

A retrospective monocentric study including patients with CRLM operated after neoadjuvant treatment. Assessment of histological response was based on the Rubbia-Brandt tumor regression grading score. The scores were grouped into two types of response: Response Group (R) and No Response Group (NR).

**RESULTS:**

The study included 77 patients (mean age=56 years, sex ratio=1.57). Node metastases were noticed in 62% of cases. Synchronous liver metastasis was present in 42 cases (55%) and metachronous liver metastasis in 45%. Neoadjuvant treatment consisted of CT only in 52 patients (68%) and CT with targeted therapy in 25 patients (32%). Chemo-induced lesions were present in 44 patients (57%). Histological response was presented (Group R) in 36 cases (47%) and absent (Group NR) in 41 cases (53%). The overall survival of our patients was 32 months. For Group R, survival was significantly greater (p=0.001). The predictive factors of histological response identified were delay in the onset of liver metastasis greater than 14 months (p=0.027) and neoadjuvant treatment combining CT and targeted therapy (p=0.031). In multivariate analysis, the type of neoadjuvant treatment (p=0.035) was an independent predictive factor of histological response.

**CONCLUSIONS:**

Predictive factors of histological response would allow us to identify patients who would benefit most from neoadjuvant treatment. These patients with CRLM onset of more than 14 months and treated with CT combined with targeted therapy would be the best candidates for a neoadjuvant CT strategy followed by surgical resection.

## RESUMO

**RACIONAL:**

A cirurgia, após a quimioterapia neoadjuvante (QT), melhora o prognóstico das metástases hepáticas colorretais (MCRC).

**OBJETIVOS:**

Avaliar os fatores preditivos da resposta histológica das metástases hepáticas colorretais (MCRC) após o tratamento neoadjuvante.

**MÉTODOS:**

Estudo retrospectivo monocêntrico incluindo pacientes com MCRL operados após tratamento neoadjuvante. A avaliação da resposta histológica foi baseada no escore de regressão tumoral Rubbia-Brandt TRG. Os escores foram agrupados em dois tipos de resposta: Grupo de resposta (R) e Grupo sem resposta (SR).

**RESULTADOS:**

Foram incluídos no estudo o total de 77 pacientes (idade média=56 anos, proporção entre os sexos=1,57). Metástases nodais foram observadas em 62% dos casos. Metástases hepáticas sincrônica estavam presente em 42 casos (55%) e metástases hepáticas metacrônicas em 45%. O tratamento neoadjuvante consistiu apenas de quimioterapia em 52 pacientes (68%) e quimioterapia com terapia direcionada em 25 pacientes (32%). As lesões induzidas por quimioterapia estavam presentes em 44 pacientes (57%). A resposta histológica estava presente (GrupoR) em 36 casos (47%) e ausente (Grupo SR) em 41 casos (53%). A sobrevida geral foi de 32 meses. A sobrevida foi significativamente maior (p=0,001) para o Grupo R. Os fatores preditivos de resposta histológica identificados foram atraso no aparecimento das metástases hepáticas superior a 14 meses (p=0,027) e tratamento neoadjuvante combinando quimioterapia e terapia direcionada (p=0,031). Na análise multivariada, o tipo de tratamento neoadjuvante (p=0,035) foi um fator preditivo independente da resposta histológica.

**CONCLUSÕES:**

Os fatores preditivos de resposta histológica permitiriam identificar os pacientes que mais se beneficiariam do tratamento neoadjuvante. Esses são os pacientes com metástases hepáticas de início há mais de 14 meses e tratados com quimioterapia combinada com terapia direcionada, os quais seriam os melhores candidatos para uma estratégia de quimioterapia neoadjuvante, seguida de ressecção cirúrgica.

## INTRODUCTION

Colorectal cancer (CRC) represents the first digestive cancer and the second cause of death by cancer in the world^21^. The liver is the main site of CRC metastasis, occurring in 50% of cases. Historically, CRC with liver metastases (LM) was associated with a poor prognosis, as the median survival rate was less than 1 year.

Today, improved survival rates have been achieved with the help of chemotherapy (CT), targeted therapies, and surgical resection of colorectal liver metastases (CRLM)^4,22^. Neoadjuvant CT aims to induce a pathological response to LM but exposes patients to liver damage with an increased postoperative risk of morbidity and mortality^1,6,9^.

Nevertheless, two-thirds of patients undergoing surgical resection of metastases develop a recurrence of their disease^11^. The histological response (HR) of CRLM after neoadjuvant treatment is a good prognostic factor documented in several studies. HR is assessed using tumor regression grading (TRG)^19,24^.

Predictive factors of HR would help in selecting patients who will benefit the most from neoadjuvant treatment to improve management and thus survival^7,8,26,29^.

The aim of our study was to identify the predictive factors of HR of CRLM after neoadjuvant treatment in metastatic CRC patients.

## METHODS

A retrospective, descriptive, monocentric study was conducted involving CRC patients with LM who underwent surgical resection after neoadjuvant treatment.

Cases were collected at the pathology department of a University Hospital Center in Tunis (Northern Tunisia) between July 2017 and July 2023. The following were not included in this study: the patients with resected LM without neoadjuvant treatment or with LM and cancer involving another organ. The following were excluded from this study: patients treated with hepatic intra-arterial CT or percutaneous radiofrequency and patients whose hospital records were not usable or whose data were missing.

Epidemiological data were collected concerning primary CRC, LM, types of neoadjuvant treatment, radiological and pathological response of LM, and survival. Data from pathology reports and review of pathology slides enabled us to specify the characteristics of the primary CRC (histological type according to the 2019 WHO classification, tumor differentiation, tumor stage, or pTNM)^2,17^.

LM data included radiological data for evaluation of tumor response according to RECIST criteria on CT or MRI, pathological features, and HR according to the TRG of Rubbia-Brandt et al. (five grades grouped into two groups: response (R) for TRG 1, 2, and 3 and no response (NR) for TRG 4 and 5)^16,19^. All statistical tests were performed with a significance level of 5%. The study was approved by the Ethics Committee of the Institution (28/2023).

## RESULTS

A total of 77 patients were included in our study, with a mean age of 56 years. Our population comprised 47 male patients (61%) and 30 female patients (39%). The primary CRC site was colonic in 57 patients (74%) and rectal in 20 patients (26%). Two histological types were identified: adenocarcinoma (ADK) without other specifications in 69 cases (90%) and mucinous colloid in eight cases (10%).

Among ADKs without other specifications, 43 cases (56%) were well differentiated, and 26 cases (34%) were moderately differentiated. Sixty of our patients (78%) were classified T3 according to the TNM classification. Lymph node metastases were noted in 48 cases (62%). Distant metastases were detected in 42 patients (55%); all of them were hepatic.

A total of 35 patients presented with metachronous LM (45% of cases), with a mean time to onset of 22 months. In total, 59 of our patients (77%) were at advanced stage (III and IV) at the time of CRC diagnosis.

Neoadjuvant treatment of LM was CT alone for 52 patients (68%) and CT plus targeted therapy for 25 patients (32%). The FOLFOX (folinic acid, fluorouracil and oxaliplatin) protocol alone was administered to 41 patients, and in combination (CT and/or targeted therapy) for 30 patients. The FOLFIRI (Folinic acid, fluorouracil and irinotecan) protocol, always associated with other drugs, was administered to 18 patients. Six patients received oral capecitabine (Xeloda®). The average number of CT courses was six courses (±2, extremes 2–12 courses). Of the 25 patients who received additional targeted therapies, 13 received Bevacizumab (Avastin®) and 12 received Cetuximab (Erbitux®).

All patients underwent thoracoabdominal CT and/or abdominal or thoracoabdominal MRI for post-treatment control with CT and showed tumor regression in 19 cases (25%), stable appearance in 23 cases (30%), and progression of lesions (increase in size of nodules or appearance of secondary lesions) in 35 patients (45%).

Anatomic hepatectomy was performed in 25 cases and non-anatomic (metastasectomy or wedge resection) in 52 cases (68%). LM was unilobar in 66% of patients and bilobar in 34% of patients (26 cases). The average number of LM was 3, with a median of 2. The mean size was 25 mm (extremes from 2 to 130 mm).

**Figure 1:**
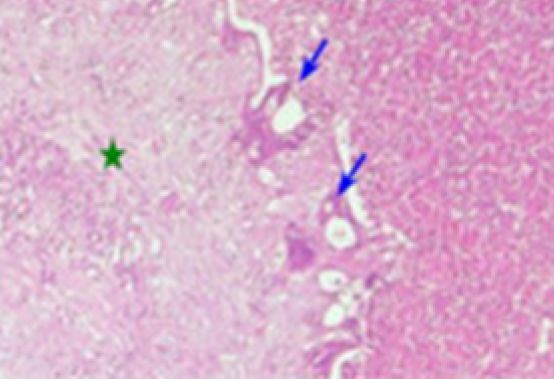
Major Response (TRG 2): Residual carcinomatous glands (blue arrows) within a predominant fibrosis (green star) (hematoxylin-eosin 25×).

**Figure 2:**
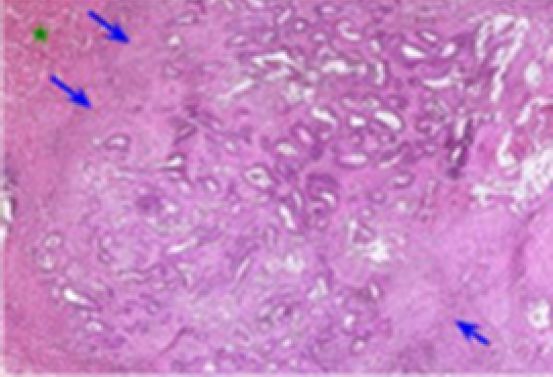
Absence of histological response (TRG 5): numerous carcinomatous glandular structures without fibrosis. Fairly large inflammatory infiltrate (blue arrows) at the metastasis/non-tumor liver interface (green star) (hematoxylin-eosin 10×).

The distribution of the HR of our patients according to Rubbia-Brandt TRG was: TRG 1 in 11 patients (14%), TRG 2 in 11 patients (14%), TRG 3 in 14 patients (19%), TRG 4 in 35 patients (45%), and TRG 5 in six patients (8%). By grouping our cases into two groups, we obtain: Response “R” group (TRG 1, 2, and 3) in 36 cases (47%) (Figure 1), No Response “NR” group (TRG 4 and 5) in 41 cases (53%) (Figure 2).

An R0 resection concerned 61 patients (79%). An R1 resection involved 16 patients (21%). Vascular emboli were present in six cases (8%). Ten patients (13%) had lymph node involvement. Only one patient had an endobiliary extension. Chemo-induced lesions were present in 44 patients (57%), 18 of whom had vascular lesions. An association of lesions was observed in 10 cases (13%).

The overall survival of our patients was 32 months. The median overall survival was 26 months (extremes 4–92 months). Factors associated with better overall survival were: metachronous LM (p=0.009, p<0.05), early stages of CRC (Stages I+II) (p=0.007, p<0.05), time to LM onset = 14 months (p=0.001, p<0.05), HR (R group or TRG 1, 2, and 3) (p=0.003, p<0.05), and absence of lymph node metastasis on the operated LM specimens (p=0.036, p<0.05).

Concerning the predictive factors of pathological response, in univariate analysis, a delay in onset of LM of more than 14 months (p=0.027, p<0.05), neoadjuvant treatment combining CT and targeted therapy (p=0.031, p<0.05), and the absence of lymph node metastasis on the operated LM (p=0.014, p<0.05) were predictive of a HR (Group R or TRG 1, 2, and 3). Analysis of demographic, primary cancer, and perioperative variables according to HR is represented in Table 1, and analysis of the pathological variables of LM according to HR is in Table 2.

**Table 1:** Analysis of demographic, primary cancer, and perioperative variables according to histological response.

Variables	Total n=77	Response n=36 (47%)	Non-response n=41 (53%)	p
Demographics				
Age (years)				
<57	38	17	21	0.452
=57	39	19	20	
Gender				
Male	47	19	28	0.123
Female	30	17	13	
Primary tumor				
Site				
Colon	57	30	27	0.103
Rectum	20	6	12	
Histological type				
Colloid carcinoma	8	2	6	0.178
ADK	69	34	35	
T stage				
pT1	5	3	2	0.910
pT2	60	30	30	
pT3	12	3	9	
N stage				
pN0	29	15	14	0.328
pN+	48	21	27	
M stage				
pM0	35	18	17	0.301
pM1a	42	18	24	
Stage				
Early (I–II)	18	11	7	0.830
Advanced (III–IV)	59	24	35	
Colorectal surgery				
Right colectomy	14	9	5	0.257
Left colectomy	15	9	6	
Low segmental resection	24	10	14	
Anterio resection	20	6	14	
Total colectomy	4	2	2	
Perioperative				
Time to onset of LM (months)				
<14	58	23	35	0.027
>14	19	13	6	
NAD treatment for LM				
CT + targeted therapy	25	16	9	0.031
CT	52	20	32	
N° of CT cycles				
=6	64	31	33	0.364
>6	13	5	8	
Radiological response				
Regression	19	14	5	0.079
Stable	23	15	8	
Progression	35	7	28	
Type of LM resection				
Anatomical hepatectomy	25	11	14	0.464
Metastasectomy	52	25	27	

ADK: adenocarcinoma; LM: liver metastasis; NAD: neoadjuvant; CT: chemotherapy.

**Table 2:** Analysis of the pathological variables of liver metastasis according to the histological response.

Pathological variables of LM	Total n=77	Response n=36 (47%)	Non-response n=41 (53%)	p
Site of LM				
Right lobe	35	19	16	0.329
Left lobe	16	6	10	
Bilobar	26	11	15	
N° of metastasis				
<2	29	14	15	0.310
=2	48	22	26	
Size of LM (mm)				
<20	38	20	18	0.214
=20	39	19	23	
Margins				
R0 (free from tumor)	61	30	31	0.292
R1	16	6	10	
Vascular emboli				
Yes	6	2	4	0.402
No	71	34	37	
Endobiliary extension				
Yes	1	1	0	0.468
No	76	35	41	
Lymph node metastasis				
Yes	10	1	-	-
No	67	35	32	
Chemically induced liver damage				
Yes	44	20	24	0.487
No	33	16	17	

LM: liver metastasis.

In multivariate analysis, after adjustment for confounding variables, among the three significant variables identified in univariate analysis, the type of neoadjuvant treatment and the absence of lymph node metastasis on the operated LM parts were identified as independent predictors of HR (Table 3).

**Table 3:** Multivariate analysis of predictive factors of histological response.

Variables	OR	95%CI	p
Time to onset of LM=14 months	0.303	0.101–0.912	0.290
Type of NAD treatment for LM	0.352	0.131–0.946	0.035
Metastatic lymph nodes on LM	9.844	1.181–82.081	0.013

OR: odds ratio; CI: confidence interval; LM: liver metastases; NAD: neoadjuvant.

## DISCUSSION

Few studies have focused on identifying predictive factors for the HR of LM from CRC. Among the studies dealing with this subject, several factors were analyzed^18^.

With regard to data concerning the primary colonic tumor, according to Chan et al., the number of histological non-responders was higher when the primary CRC was rectal, but there was no significant correlation (p=0.103, p>0.05)^8^. In contrast, T3 or T4 parietal invasion of CRC was considered an independent predictor of poor HR in the study by Zhang et al^31^. In our study, we found that the absence of HR was greater for advanced CRC stages (III+IV) (24 patients in the R group vs. 35 in the NR group) and similar proportions of response and non-response for early stages (11 cases in the R group vs. 7 in the NR group), but with no statistical relationship (p=0.830). As for the pTN stage of primary CRC, two studies reported a higher rate of radiation non-responders (on CT) when the CRC was N+, but with no significant correlation^8,31^. For the KRAS mutation, it was considered a good predictor of HR by some teams^15^. Yet a positive preoperative CEA (carcinoembryonic antigen) level was predictive of histological non-response (p=0.001, p<0.05) according to Cai et al. and Wang et al., although we were unable to prove this association in our study^7,29^.

Apart from data concerning primary CRC, we found that a delay in LM onset of more than 14 months was predictive of HR (p=0.027, p<0.05), but this was not an independent predictive factor. Arru et al. found that a time to onset of early LM of less than 12 months was only a negative prognostic factor for 5-year overall survival, with no association with histological response^3^.

In terms of perioperative data, multiple and bilobar LM were shown to be significantly associated with poor HR after CT^8,31^.

According to our findings, in univariate analysis, neoadjuvant treatment combining CT and targeted therapy was predictive of HR (p=0.031, p<0.05). In multivariate analysis, type of neoadjuvant treatment was an independent predictive factor of HR (OR 0.352; 95%CI 0.131–0.946; p=0.035, p<0.05). This is consistent with the results reported in the literature^12,27,31^. Klinger et al. conducted a study including 295 cases of CRLM and found that the addition of bevacizumab to neoadjuvant therapy achieved an almost threefold increase in TRG1 rates, rising from 6 to 20% in the bevacizumab group. Bevacizumab improved pathological response and patient survival, shifting tumor regression to lower grades (p=0.008, p<0.05)^14^. It has been shown that in addition to cytotoxic CT (cytotoxic T cell), anti-VEGF (anti-vascular endothelial growth fator) and anti-EGFR (anti-epidermal growth factor receptor) normalize tumor vascularization, thereby increasing the delivery of CT to tumor cells^13,25,30^.

Concerning radiological response, we found in our study that the HR rate was higher in cases of radiological regression (14 patients in group R vs. 5 in group NR) and a lower response rate in cases of progression (7 cases in group R vs. 28 in group NR), but with no statistically significant relationship (p=0.079, p>0.05). Cai et al. reported that patients with a radiological response had a more favorable pathological response than those with no radiological response (70 vs. 22.4%, p<0.001, p<0.05)^7^. Serrablo et al. have as well confirmed the correlation between radiological response, assessed by CT scan, and the HR of CRLM to neoadjuvant treatment^20^. Other teams have also confirmed this correlation by using CT scan or MRI to assess the radiological response^5,14,28^. Thus, the radiological response allows a better selection of patients eligible for surgery^14,20^.

Concerning histological data of operated LM, Wang et al. showed that resected small-diameter LM (less than 2 cm) had higher pathological response rates than larger LM (p<0.05)^29^. The presence of a peritumoral inflammatory infiltrate was associated with a good response and prognosis^23^. The high density of tumor-infiltrating lymphocytes correlates with a superior survival rate in patients with TRG 1 or 2. These results confirm the relationship between peri-tumoral lymphocyte infiltration and its impact on the efficacy of CT and targeted therapies^10,31^.

Data related to primary CRC (clinical, biological, and histological) and perioperative elements of CRLM (therapeutic and radiological) represent important prognostic factors for survival. Some are predictive of a good HR after neoadjuvant treatment of LM. These characteristics are thus tools for therapeutic choices and good indicators for the long-term evolution of CRC.

## CONCLUSIONS

Patients with LM onset of more than 14 months and treated with CT combined with targeted therapy would be the best candidates for a neoadjuvant CT strategy followed by surgical resection. To date, there are still gaps in the prognostic value of the pathological features of resected CRLM. However, they remain interesting to guide surgeons and oncologists in the subsequent post-surgical therapeutic management.
